# A rare case of severe myositis as paraneoplastic syndrome on breast cancer

**DOI:** 10.1186/s12957-015-0534-5

**Published:** 2015-04-01

**Authors:** Leonardo Pires Novais Dias, Ana Luiza Antunes Faria, Maissa Marçola Scandiuzzi, Claudia Luci dos Santos Inhaia, Jorge Yoshinori Shida, Luiz Henrique Gebrim

**Affiliations:** Department of Senology, Pérola Byington Hospital, Avenida Brigadeiro Luís Antônio 683, Bela Vista, , CEP 01317-000, São Paulo - SP, Brazil

**Keywords:** Myositis, Dermatomyositis, Breast cancer, Paraneoplastic syndrome

## Abstract

**Background:**

Dermatomyositis and polymyositis are both types of idiopathic inflammatory myositis characterized by inflammation and weakness of proximal skeletal muscles and skin rash.

**Case:**

A 49-year-old Caucasian woman recently diagnosed with breast cancer classified as T1N2M0, stage IIIA, presenting skin rash associated with heliotrope and Gottron’s papules. In addition, there was a progression to a severe reduction in proximal muscle strength with severe dysphagia. The initial treatment was conducted, and the patient recovered from all symptoms and followed adjuvant cancer management.

**Treatment:**

At first, high dose of corticosteroid was administered as pulse therapy, and a radical mastectomy was indicated due to the severe symptoms of the paraneoplastic syndrome. Then chemotherapy and radiotherapy were applied, and oral corticoid associated with immunosupressive drug was administered for dermatomyositis control.

**Discussion:**

The association between myositis and an increased risk of cancer has been demonstrated over the years. This patient has a high probability of dermatomyositis diagnosis. The initial treatment with high dose of glucocorticoids may result in an improvement of muscle lesions. Second-line treatment with azathioprine, methotrexate, or cyclophosphamide may be required for aggressive disease. Removal of the cancer induces improvement of paraneoplastic syndrome.

**Conclusion:**

Dermatomyositis can be a clinical manifestation of a paraneoplastic syndrome in patients with breast cancer. It is a rare diagnosis, and there is little evidence to guide treatment until now. It is possible to control the evolution of dermatomyositis with high doses of glucocorticoids in almost all cases; however, in severe cases of paraneoplastic syndrome, cancer treatment should start immediately.

## Background

Dermatomyositis (DM) and polymyositis (PM) are both types of idiopathic inflammatory myopathies (IIM) characterized by inflammation and weakness of proximal skeletal muscles. DM disease is presented with specific cutaneous signs. Myositis can affect various organs with extra muscular manifestations: lungs, heart, joints, and intestine [1-3]. Muscle inflammation and weakness are the key features of this myopathy [4].

The reported incidence for DM varies from 0.5 to 0.89 per 100,000/year [3], affecting mostly middle-aged women in a 2:1 ratio with men in the same age group. In general, it is described as idiopathic, but malignancies associated with myopathies have been extensively reported in the medical literature since 1916 and then confirmed by subsequent meta-analyses [5,6].

The type of malignancy generally reflects those found in age and sex matched populations. Lung and colorectal cancers were the most common cancers in men from Western country cohorts; however, among women, 20% is related to breast neoplasia [5,6].

The authors of this study reported a case of breast cancer in an unusual presentation in order to arouse the attention of professionals, guide diagnosis and establish appropriate management in severe cases.

## Case presentation

A 49-year-old Caucasian woman with a tumor in the left axilla came to evaluation at the Pérola Byington Hospital (PBH) in São Paulo, Brazil. The ultrasound revealed an irregular breast nodule measuring 1.8 cm, suspected of malignancy, in the lower medial quadrant of the left breast, and an axillary lymph node of 3.5 cm, clinically matted. Both underwent core needle biopsy, and the diagnosis was invasive carcinoma of no special type, histological, and nuclear grade III and II, respectively. The tumor was positive for hormone receptors (estrogen and progesterone), Her2/neu +3/+3 and Ki-67 40%. According to the first evaluation, the cancer stage on TNM classification was IIIA (cT1cN2cM0) [7]. The screening for metastasis was negative.

**Figure 1:**
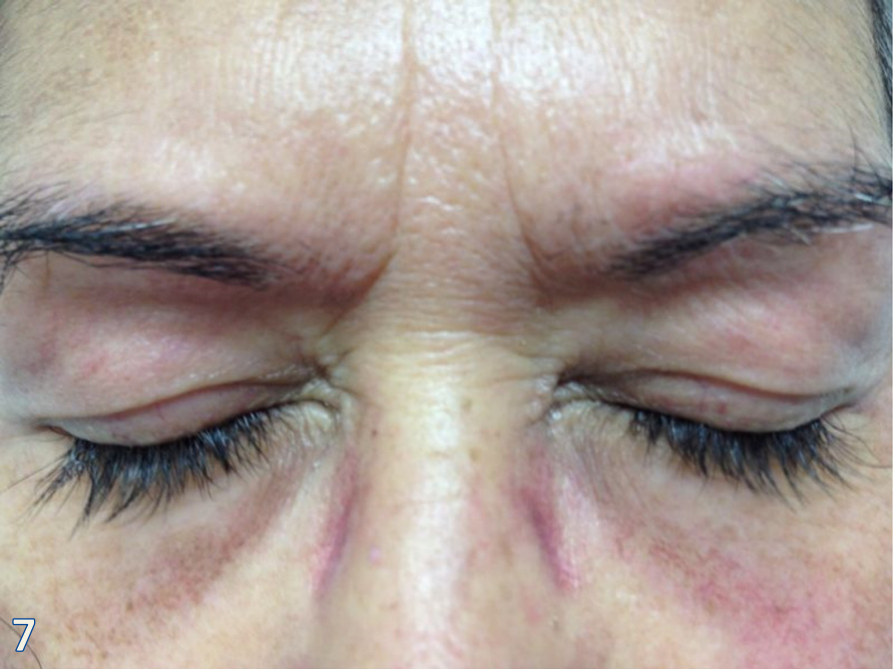
**Heliotrope.**

**Figure 2:**
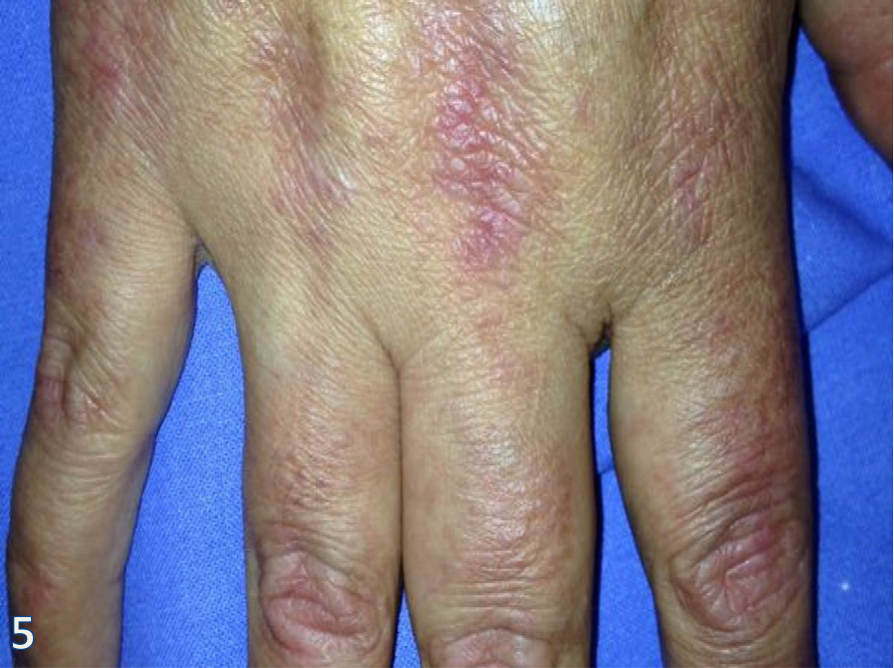
**Gottron’s papules.**

On the following month, the patient developed a characteristic group of signs and symptoms. First appeared was erythema on the face, trunk, and extensor areas of the limbs. Also, heliotrope and Gottron’s papules were identified (Figures 1 and 2). The heliotrope eruption consists of a violaceous or dusky erythema with edema in a periorbital distribution. When it occurs bilaterally, it may be a subtle skin finding as it often only involves the upper lid. Gottron’s papules are found over bony prominences, particularly the knuckles. They consist of slightly elevated, violaceous to dusky red papules or plaques that may develop a poikilodermatous appearance and may become atrophic. These lesions can be confused with those of systemic lupus erythematosus [8].

**Figure 3:**
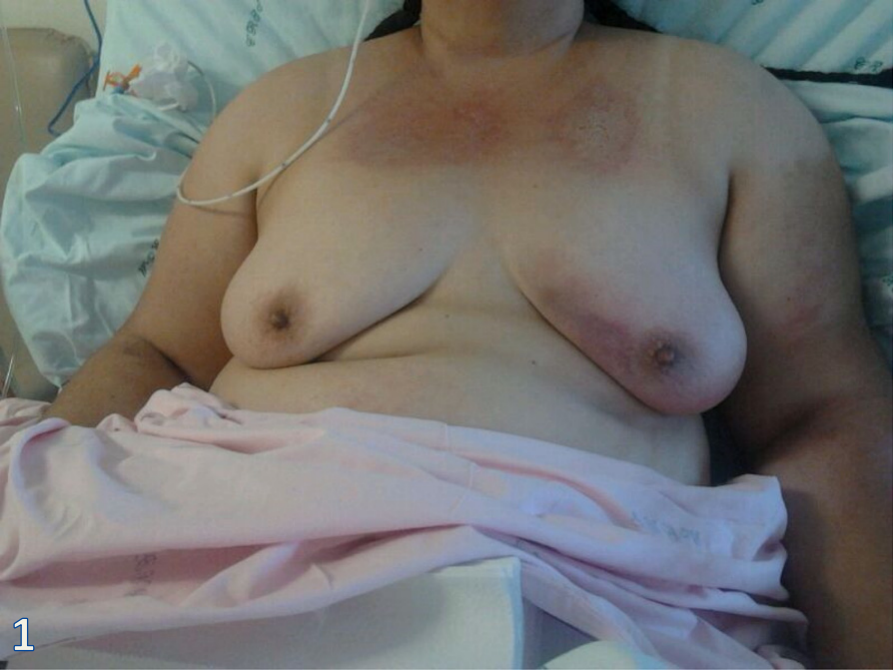
**The patient, on the second day on the ward, restricted to bed, unable to do any activity.**

One week after, a significant weakness started, followed by progressive reduction on proximal muscle strength in a symmetric distribution associated with progressive ascendant edema. On the fourth week, severe edema and dysphagia were identified. After 1 month, the patient was restricted to bed, unable to perform any daily activity, and she was brought to be evaluated in PBH (Figure 3). During those 4 weeks, the patient was evaluated by the primary care physicians and just symptomatic medication was offered.

The patient was admitted to the ward, and the laboratory tests showed creatine kinase (CPK) of 34,875 U/L, aspartate aminotransferase of 786 U/L, alanine aminotransferase of 352 U/L and lactate dehydrogenase of 4,103 U/L. The high clinical suspicion of DM and the severity of the condition guided the medical team to initiate the treatment immediately.

It is well known that CPK is a toxic substance for the kidney, and in order to prevent kidney acute lesions, it was offered an intravenous great amount of physiologic solution 0.9%, aiming a diuresis of 300 ml/h or more and a glicophysiological solution 5% with sodium bicarbonate, to reduce urine acidosis, associated or not with mannitol [9]. High dose of corticosteroid was administered as pulse therapy, using methylprednisolone 1 g/day, for three consecutive days [8]. After the first dose of pulse therapy, the patient related that the progression of the muscle fatigue stopped. According to NCCN guideline, a cancer stage IIIA should be treated with preoperative chemotherapy [10], but considering the clinical suspicious of DM as a paraneoplastic syndrome, some papers describe faster recovery of the dermatomyositis symptoms if the resection of the cancer is realized prior to the chemotherapy [11-14]. A medical meeting decided for radical mastectomy with axillary dissection, and the procedure was performed on the following week.

**Figure 4:**
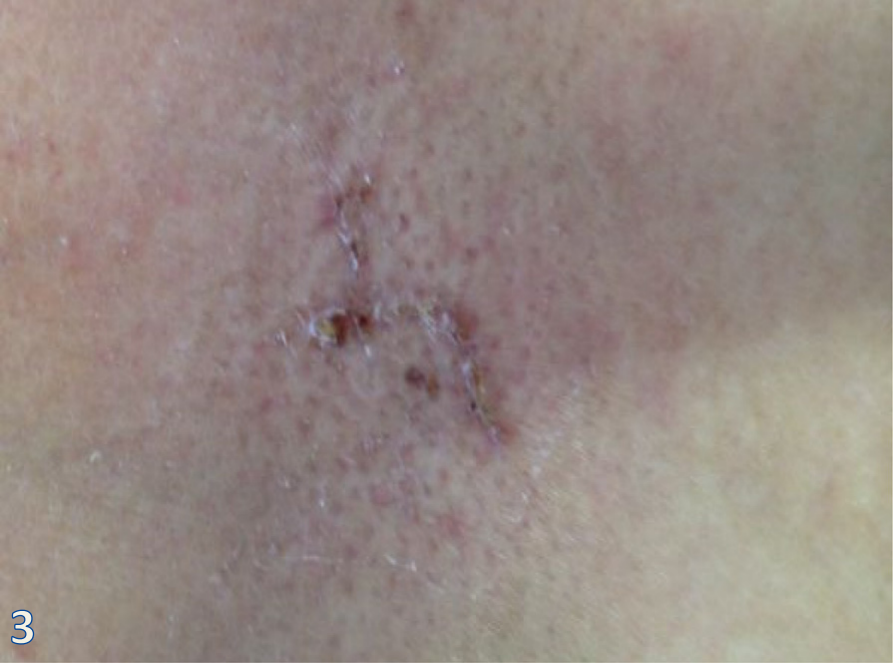
**Skin rash 1 week after surgery.** Improvement of the lesion after the resection of the tumor mass is remarkable.

The histopathological examination revealed primary tumor of 2.0 cm, with metastasis in 8 of 16 axillary lymph nodes. Also, the histopathological cutaneous biopsy analysis was performed, and the skin biopsy specimen revealed a superficial perivascular lymphocytic infiltrate with vacuolar degeneration and necrotic keratinocytes along the basement membrane, epidermal hyperplasia with focal areas of atrophy, and compact orthokeratosis (Figure 4).

**Figure 5:**
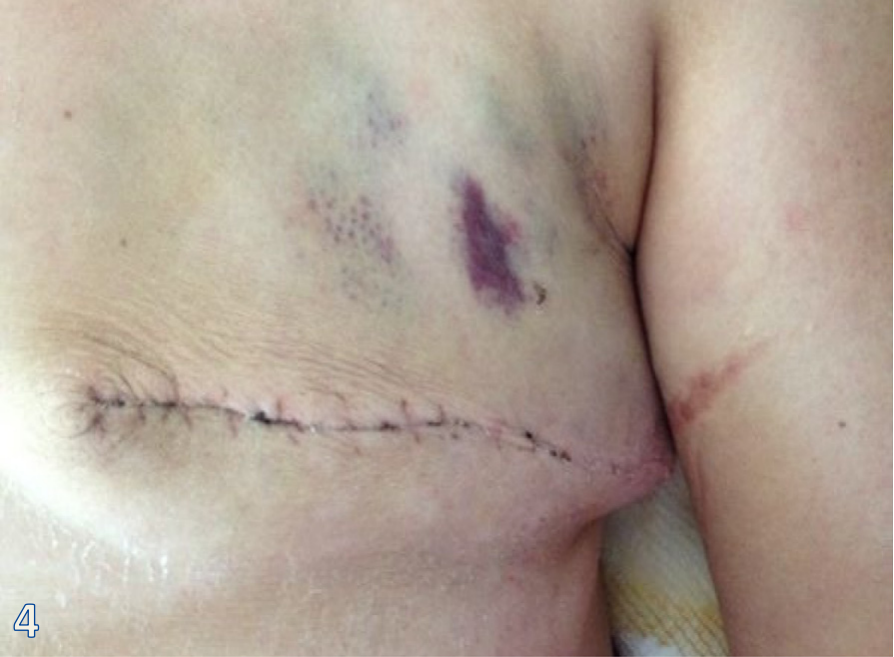
**Skin rash 2 weeks after surgery.** There is an almost complete remission of the lesion.

There were no postoperative complications, and after 2 weeks, full remission of the edema and gradual recovery of muscle strength and swallowing capacity were evident (Figure 5). In the third week, the patient was already able to walk with assistance and was discharged to follow up with the team of clinical oncology.

On the follow-up, the patient was submitted to adjuvant treatment: first, chemotherapy with 6 cycles of cyclophophamide (500 mg/m^2^), doxorubicin (60 mg/m^2^), and paclitaxel (175 mg/m^2^) and a rest period of 21 days, followed by trastuzumab with initial dose of 8 mg/kg and maintenance dose of 6 mg/kg, for 18 cycles, with 21 days of rest period.

Second, after concluding the first 6 cycles, the patient was submitted to 25 radiotherapy sessions on the chest wall and supraclavicular fossa, with a total of 50 Gy (2 Gy/day).

Third, the patient was evaluated and verified that she was on menopause, and an aromatase inhibitor was initiated. The first choice was anastrozole, on the dose of 1 mg/day, during 5 years.

On the other hand, the treatment of the paraneoplastic syndrome was maintained with oral corticoid, initially with 1 mg/kg of prednisone. After 1 year of follow-up, we introduced azathioprine, 2 mg/kg oral daily, and the oral corticoid was reduced to 10 mg. On two occasions, the medical team suspended the corticoid, and after a few days, the skin rash appeared again. So, after rechecking the literature, our group decided to keep the corticoid on the lowest dose possible, and 10 mg is the minimum that the patient accepts with no symptoms [8,15,16].

Due to the chronic use of oral corticoid associated to anastrozole, the medical team introduced oral calcium carbonate plus vitamin D [17].

The last screening for metastasis, after 18 months of follow-up, was negative and the DM was controlled.

## Discussion

The association between IIM and cancer has been extensively studied in adults. Many epidemiological studies demonstrated this association, which appears stronger for DM than for PM. The first case suggesting an association between cancer and DM was reported in 1916. At present, the reported incidence of cancer association with DM varies widely, from less than 7% to over 30% [18-21]. One paper has reported that malignancy may precede myopathy by 2 years [22], while another have described neoplasias in DM even after 5 years of disease [23]. Thus, ovarian cancer or breast cancer in females and lung cancer in males are the main malignancies associated with DM [3,18,19].

**Figure 6:**
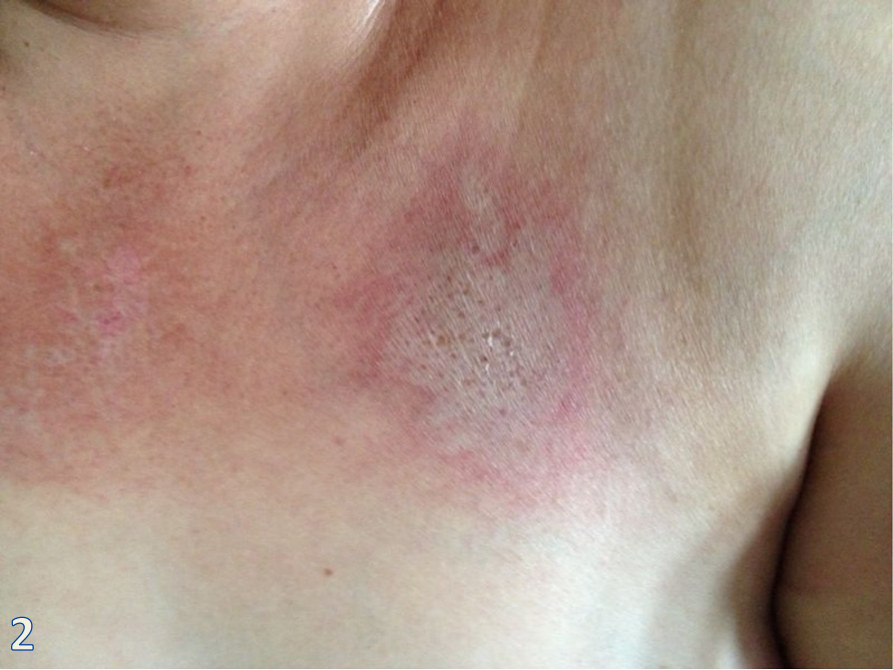
**Typical skin rash, with atrophic areas on the trunk.**

Dermatomyositis diagnosis was based on Bohan and Peter’s criteria, and the patient should have a typical rash and at least three of following manifestations: symmetric proximal muscle weakness, muscle biopsy evidence of myositis, increase in serum skeletal muscle enzymes, and characteristic electromyographic pattern. The patient in this case study had the typical skin rash (Figure 6) and two other manifestations related to DM, with high clinical suspicious of the diagnosis [24].

Other symptoms can occur and may include pitting edema, dysphagia secondary to bulbar muscle weakness, and nasal regurgitation of liquids or aspiration pneumonia and dyspnea [11].

Patients with DM have an increased incidence of human leukocyte antigen (HLA)-B8 and HLA-DR3. There is a significant association between patients with myositis and antibodies to histidyltransfer RNA synthetase (Jo-1antigen), which has been linked to the HLA-DR3 antigen. Anti-Jo-1antibody positive patients may have an increased risk for the development of interstitial pulmonary disease [8]. In addition, the possible autoantibodies related were not assessed in this study.

The management of DM/PM is based on diagnostic accuracy, assessment of disease activity prevention, patient education, and psychosomatic support [8]. Despite the lack of evidence from controlled trials to guide treatment, corticosteroids remain the agents of choice for inflammatory myopathy [15]. Patients with DM treated with high-dose prednisone generally have a good initial response, with rates of initial remission varying from 27% to 87%. Not all types of myositis, however, respond favorably to corticosteroids, and some authors initially add a first-line immunosuppressive agent in combination with high-dose corticosteroids in severe cases of myositis [15,16]. Indeed, immunosuppressive agents are both steroid sparing and effective, serving to mitigate corticosteroid-related side effects and at the same time treating the aforementioned serious extra muscular manifestations, although there is a need to weigh the increased risks of immunosuppression [15].

Methotrexate may be successfully used as a corticosteroid sparing agent in the treatment of DM. A therapeutic effect may not be seen for 4 to 8 weeks. The recommended oral dose is 5 to 20 mg/week. Patients must be monitored for hepatotoxicity, renal insufficiency, and pulmonary fibrosis. Azathioprine is an alternative agent effective at doses of 2 to 3 mg/kg/day in divided doses, with a maintenance dose of 0.5 mg/kg/day. Hematologic monitoring is essential to detect leukopenia and anemia [8].

Treatment with high dose of glucocorticoids may result in an improvement of CPK levels within the first couple of weeks. There is often a delay before muscle strength recovers, at that time, standard breast cancer therapies are recommended. In patients with breast cancer and DM, the role of neoadjuvant chemo/hormonal therapy is debatable, and there is no solid data available in this setting, and the response can be slower than with surgical treatment [11,12,25].

Age, severity of muscle disease, and systemic involvement all affect the prognosis of patients with DM and PM. Studies have shown a poor outcome in patients with pulmonary fibrosis and dysphagia. Rapid response to corticosteroid therapy has been associated with a favorable prognosis [8].

## Conclusion

DM can be a clinical manifestation of a paraneoplastic syndrome in patients with breast cancer. It is a rare diagnosis, and there is little evidence to guide treatment until now. It is possible to control the evolution of DM with high doses of glucocorticoids associated with immunosuppressive agents and treat cancer with surgery as soon as possible [13,14]. Removal of the cancer induces improvement of paraneoplastic syndrome [12].

## Consent

Written informed consent was obtained from the patient for publication of this case report and any accompanying images. A copy of the written consent is available for review by the Editor in Chief of this journal.

## Abbreviations

DM: dermatomyositis
PM: polymyositis
IIM: idiopathic inflammatory myopathies
PBH: Pérola Byington Hospital
CPK: creatine kinase
